# Effects of supplementing diet with Thyme (*Thymuas vulgaris* L.) essential oil and/or selenium yeast on production performance and blood variables of broiler chickens

**DOI:** 10.1002/vms3.736

**Published:** 2022-01-25

**Authors:** Shokufe Noruzi, Mehran Torki, Hamed Mohammadi

**Affiliations:** ^1^ Animal Science Department College of Agriculture and Natural Resources Razi University Kermanshah Iran; ^2^ Department of Agriculture Payame Noor University Tehran Iran

**Keywords:** broiler, glutathione peroxidase, herbal and natural additives, organic selenium, performance, thyme (Thymuas vulgaris L.)

## Abstract

**Background:**

Organic products for animals are becoming more widely accepted by consumers. Using herbal additives may lead to more healthy animal products. In this research, it is hypothesized that thyme essential oil (TEO) and/or selenium yeast (SY) would be helpful to enhance production performance in broilers.

**Objective:**

In the current study, the effects of adding TEO and/or SY to the diet on broiler performance and blood parameters were evaluated in broiler chickens.

**Methods:**

A total of 480 chicks were distributed in 24 cages with 20 chicks (10 males and 10 females) each and assigned to be fed four iso‐caloric and iso‐nitrogenous diets including two levels (0 and 250 mg/kg) of TEO and two levels (0 and 0.3 mg/kg) of SY in a 2 × 2 factorial arrangement with six replicates.

**Results:**

Adding SY significantly decreased feed intake in finishing period (22–42 d) (*p* < 0.05). Supplementation with SY and TEO significantly decreased plasma uric acid and triglyceride levels, respectively (*p* < 0.05). However, neither of the supplements had any influence on the plasma glucose and albumin levels (*p* > 0.05). The lowest level of plasma cholesterol was detected in the birds fed the TEO‐supplemented diet (*p* < 0.05). Addition of SY significantly increased blood glutathione peroxidase activity (*p* < 0.05).

**Conclusions:**

Dietary supplemental TEO has a favourable effect on feed intake, weight gain, and body weight values. Besides, SY may decrease blood concentration of uric acid, as well as blood glutathione peroxidase activity. An interaction is detected between TEO and SY on blood cholesterol.

## Graphical abstract

Dietary supplemental thyme oil has a favorable effect on feed intake, weight gain, and body weight values; in addition, Se may decrease blood concentration of uric acid, as well as blood glutathione peroxidase activity. An interaction is detected between thyme oil and Se on blood cholesterol.

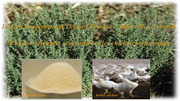

## 1 INTRODUCTION

Recently, antibiotics or growth promoters have been banned to prevent the development of antibiotic resistance in human pathogenic bacteria and to remove antibiotic residues from poultry products. On the other hand, there is increasing public and government pressure in several countries of the European Union (EU) and some non‐EU nations to search for natural alternatives to antibiotics (Attia et al., 2019; McCartney, 2002; Williams & Losa, 2001). This reality has led to a new urgency in the search for antibiotics (as growth promoters) replacements. Many aromatic plants have been used for medical purposes since the pre‐historic times (Edens, 1996). Use of various plant materials as dietary supplements may positively affect animal health and productivity (Attia et al., 2017a, 2019). A large number of active compounds in these supplements may, therefore, present a more acceptable defence against bacterial attack than chemical anti‐microbial compounds (antibiotics). There is evidence to suggest that herbs, spices and various plant extracts have appetizing and digestion‐stimulating properties and anti‐microbial effects (Alcicek et al., 2003; Attia et al., 2019; Langhout, 2000; Surai, 2000; Youdim & Deans, 2000), which stimulate the growth of beneficial bacteria and minimize pathogenic bacterial activity in bird gastrointestinal tract (Watanabe et al., 1997). On the other hand, supplementing the diet with the plant material that is rich in active substances with beneficial effects for the immune system can be used as an alternative to antibiotic growth promoters.

Several studies have reported the beneficial effects of thyme in poultry nutrition (Al‐Kassie, 2009; Attia, 2017a, 2018; Demir et al., 2005; Demir & Kilinc, 2008; El‐Ghousein & Al‐Beitawi, 2009; Najafi & Torki, 2010). Thyme (*Thymus vulgaris*), an aromatic plant, belongs to the Lamiaceae family. The main components of thyme are phenols, thymol (40%) and carvacrol (15%) (Azaz et al., 2004), which are the main anti‐bacterial active substances (Demir et al., 2005). A wide variety of pharmacological activities are detected for thymol such as anti‐spasmodic, anti‐oxidant, anti‐microbial, anti‐cancer, anti‐viral, anti‐inflammatory and growth enhancer (Abd El‐Hack et al., 2016). Thyme is also used traditionally for several medicinal purposes, namely respiratory disease, anti‐microbial, anti‐nociceptive, etc. (Demir et al., 2005; Demir & Kilinc, 2008). Ground thyme has been shown to inhibit the growth of *Salmonella typhimurium* when added to the media (Aktug & Karapinar, 1986). The essential oil of the thyme has been shown to inhibit the growth of *Escherichia coli* in the media (Marino et al., 1999). Bolukbasi and Erhan (2007) showed that supplementation of 0.1 and 0.5% thyme to layer diets improved feed conversion ratio (FCR) and egg production and also reduced *E. coli* faecal content (Bolukbasi & Erhan, 2007).

Selenium was discovered in 1817 and it is essential for normal life processes (Kohrl et al., 2000), and all animals, including poultry, need this inorganic element (Attia et al., 2010). Definite roles of Se in immune function, health and productivity are demonstrated. Broilers need 0.15 mg of Se/kg of diet during growth period (National Research Council, 1994), which often is met by natural ingredients in diets based on types of soil; however, inorganic Se (as sodium selenite [Na_2_SeO_3_]) is usually added to commercial diets to guarantee the broilers will not experience with Se deficiency symptoms. Organic Se in the yeast has been introduced as a probable alternative for Na_2_SeO_3_ (Payne & Southern, 2005). The biologically active form of Se was found in 1973, when glutathione peroxidase was identified as a very potent antioxidant protecting the cell from damage due to oxidation by free radicals (Rotruck et al., 1973). The activity level of this enzyme in the liver or plasma is indicative of Se supply level to the organism. Glutathione peroxidase destroys hydrogen peroxide and hydroperoxides to protect cells and membranes from oxidative damage (Tucker, 2002).

The objective of this study was to evaluate the effects of adding thyme essential oils (TEO) and/or SY on performance and some blood parameters of broiler chickens. Surveying any probable interaction between TEO and SY may be mentioned as a novelty and necessity of the current study.

## 2 MATERIALS AND METHODS

### 2.1 Animals, treatments, experimental design and management

A total of 480, day‐old Ross‐308 broiler chicks (body weight [BW] 42 ± 5 g) were purchased from a commercial hatchery and housed in 24 identical cages providing 0.13 m^2^ per bird. Each cage was equipped with two trough feeders and two nipple drinkers. The chicks were randomly allocated to four dietary treatments with six replicates with 20 birds each (10 males and 10 females), in a completely randomized design and a 2 × 2 factorial arrangement. The rearing house had windows and was equipped with a smart ventilation system and heating systems controlled by a thermostat. The relative humidity was kept as close as possible to a constant 55%. The birds were reared according to the temperature schedule recommended in Ross 308 management handbook. Feed and water were offered ad libitum and feed intake (FI) and BW were recorded for the total period. The lighting schedule was 23‐h light/1‐h dark cycle with an average light intensity of 15 lx, which was maintained until the end of the experiment. Daily probable mortalities were recorded and used to correct performance criteria. Four iso‐caloric and iso‐nitrogenous experimental diets (in mash form) containing supplemental SY (0 and 0.3 mg/kg diet) and thyme (0 and 250 mg/kg diet) were formulated according to National Research Council 1994 (Table 1) and offered to the chickens during two experimental periods (11 to 21 and 22 to 42 d of age). The basal diet (with no SY and TEO) was offered from 1 to 10 days of age. Mortality rate throughout the experimental period was very low (only one), and those data were not subjected to analysis.

**TABLE 1:** Composition and calculated analysis of the experimental diet, %

Ingredient composition	Growing period (11–22 d)	Finishing period (22–42 d)
Corn	55.86	64.50
Soybean meal	38.45	28.71
Soybean oil	1.76	2.77
Oyster shell	0.92	0.94
Dicalcium phosphate	1.85	1.96
Common salt	0.31	0.31
Sodium bicarbonate (NaHCO_3_)	0.08	0.10
Vitamin premix^†^	0.25	0.25
Mineral premix^‡^	0.25	0.25
DL‐Methionine	0.19	0.21
Lysine‐HCL	0.08	0

**Nutrient composition (as fed basis), %, unless otherwise stated**		
Dry matter	89.5	89.8
Metabolisable energy (Kcal/kg)	2950	3000
Crude protein	21.00	18.00
Crude fiber	3.93	3.19
Ether extract	4.11	4.49
Calcium	0.9	0.85
Available phosphorus	0.45	0.42
Sodium	0.22	0.16
Chloride	0.22	0.22
Methionine + Cystine	0.85	0.76
Lysine	1.12	0.97

^†^ Vitamin mixture per 2.5 kg of diet provides the following: vitamin A, 7.700.000 IU; vitamin D_3_, 3.300.000 IU; vitamin E, 6.600 mg; vitamin K_3_, 550 mg; thiamine, 2.200 mg; riboflavin, 4.400 mg; vitamin B6, 4.400 mg; Ca pantothenate, 550 mg; nicotinic acid, 200 mg; folic acid, 110 mg; choline chloride, 275.000 mg; biotin, 55 mg; vitamin B_12_, 8.8 mg.

^‡^ Mineral mixture per 2.5 kg of diet provides the following: Mn, 66.000 mg; Zn, 66.000 mg; Fe, 33.000 mg; Cu, 8.800 mg; Se, 300 mg; I, 900 mg.

### 2.2 Feed additives

Selenized yeast (Sel‐Plex Alltech Inc.), as a source of organic Se for broiler production, was recently approved for use in the United States. It contains at least 50% Se‐Met; this form of Se is readily available and is actively absorbed from the intestine via the Na^+^‐dependent neutral amino acid pathway (Edens, 2001). Supplemental thyme (*Thymus vulgaris* L.) essential oil which was purchased from Barij Essence Pharmaceutical Co., Kashan, Iran, contained thymol (45.1%), para‐cymene (22.5%), gamma‐terpinene (8.3%) (Mehran et al., 2016). The basal diet was supplemented with these supplements.

### 2.3 Performance and blood parameter determination

BW, weight gain (WG), FI and FCR were determined via a digital scale (±1 g) throughout growing, finishing and overall periods (11 to 21, 22 to 42 and 11 to 42 d of age, respectively). Plasma levels of glucose, albumin, cholesterol, uric acid and triglycerides were determined on day 42 of age. After a 15‐h fasting period, blood samples (3 mL) were collected in non‐heparinized tubes at 42 days of age from one bird in each cage (*n = 24*) by puncturing the brachial vein and centrifuged at 2000 × *g* for 10 m to obtain serum (SIGMA 4–15 Lab Centrifuge, Germany). Individual serum samples were analysed for glucose, albumin, cholesterol, uric acid and triglycerides with a spectrophotometer using commercially available kits (Pars Azmun, Tehran, Iran). Blood GPx activity was assessed by measuring the oxidation of NADPH to NADP^+^ at 340 nm (Paglia & Valentine, 1967), using commercial (Pars Azmun, Tehran, Iran). Briefly, 200 μL of serum sample was incubated with 0.2 M phosphate buffer (pH 7.2), 15 mM GSH and 50 units of glutathione reductase for 15 min at 37°C. Following the addition of 1.5 mM NADPH and 2 mM H_2_O_2_, a reduction in the absorbance was measured at 340 nm using a spectrophotometer (Hitachi U‐2001, Tokyo, Japan). One unit of GPx activity was defined as the amount of enzyme that oxidizes 1 μmol NADPH/min.

### 2.4 Statistical analysis

All of the parameters were analysed as follows: *Y*_ijk_ = μ + Th_i_ + SY + ThSe_ij_ + e_ijk_; where *Y*_ijk_ is the measured parameter, *μ* is the overall mean, *Th_i_* is the main effect of the thyme, SY_j_ is the main effect of the SY, ThSe_ij_ is interaction between thyme and SY and *e_ijk_* is the residual error. Each cage (containing 20 chicks) was supposed as an experimental unit. Where the interaction effect was significant, the effects of the main factors were not considered. Data were analysed using the General Linear Model procedure of SAS (2001). Tukey's multiple range test was used to detect the differences (*p* < 0.05) among different group means (*p* < 0.05).

## 3 RESULTS

### 3.1 Production performance

Table 2 shows the effect of TEO and SY on the performance of the broiler chickens. SY addition decreased FI during the finishing period (*p* < 0.05). Neither thyme nor SY supplementation had a significant effect on WG, BW and FCR values of the broiler chickens (*p* > 0.05).

**TABLE 2:** The effects of dietary supplemental thyme and/or Se on feed intake (FI, g/bird per day), weight gain (WG, g/bird per day), body weight (BW) and feed conversion ratio (FCR) of the broiler chickens

		FI (g)			WG (g)			BW (g)		FCR (g:g)		
Groups		Growing period (11–22 d)	Finishing period (22–42 d)	Overall period	Growing period (11–22 d)	Finishing period (22–42 d)	Overall period	Growing period (11–22 d)	Finishing period (22–42 d)	Growing period (11–22 d)	Finishing period (22–42 d)	Overall period
Thyme												
0		71.3	172	121	45.5	86.2	65.8	500	1810	1.58	1.99	1.79
250		72.4	168	120	48.6	90.4	69.5	535	1898	1.50	1.87	1.68
Se												
0		72.0	174^a,b^	123	46.9	88.4	67.7	520	1858	1.55	1.98	1.76
0.3		71.7	165^a,b^	119	47.2	88.1	67.7	516	1851	1.53	1.89	1.71
Thyme	Se											
0	0	72.0	178	125	45.2	85.6	65.4	497	1797	1.60	2.10	1.84
250	0	70.8	165	118	45.8	86.8	66.3	504	1823	1.55	1.90	1.73
0	0.3	72.1	170	121	48.7	91.3	70.0	535	1917	1.49	1.87	1.68
250	0.3	72.6	166	120	48.6	89.4	69.0	534	1878	1.50	1.88	1.69
SEM		1.95	3.09	2.53	2.38	4.09	3.25	22.4	100	0.90	1.10	0.85
*p*‐Values												
Thyme		0.357	0.302	0.516	0.055	0.117	0.061	0.070	0.117	0.213	0.102	0.083
Se		0.753	0.012	0.066	0.832	0.905	0.999	0.832	0.905	0.756	0.237	0.349
Thyme × Se		0.443	0.123	0.099	0.812	0.553	0.576	0.812	0.553	0.641	0.184	0.263

^a,b^ Means within a column followed by the same superscript are not significantly different (*p* > 0.05).

SEM, Standard error of mean.

### 3.2 Blood variable

Tables 3 and 4 show the effects of TEO and SY on blood variables and blood GPx activity at the end of the experimental period (42 d of age). The supplementation with SY and/or TEO did not influence glucose and albumin levels (*p* > 0.05). SY significantly decreased uric acid levels. Besides, triglyceride levels significantly decreased in TEO group compared to the control group at the end of the experimental period (d 42) (*p* < 0.05). The supplementation with SY significantly increased blood GPx activity at the end of the experimental period (*p* < 0.05).

**TABLE 3:** The effects of dietary supplemental thyme and/or selenium on blood biochemical parameters of the broiler chickens

Groups		Glucose, mg/dL	Albumin, mg/dL	Uric acid, mg/dL	Cholesterol, mg/dL	Triglycerides, mg/dL
Thyme						
0		242	2.82	3.74	132	135^a,b^
250		240	2.90	3.69	126	125^a,b^
Se						
0		242	2.75	4.07^a,b^	128	131
0.3		242	2.97	3.37^a,b^	129	128
Thyme	Se					
0	0	244	2.87	4.28	143^a,b^	140
250	0	239	2.72	3.86	114^a,b^	122
0	0.3	242	2.86	3.21	121^a,b^	130
250	0.3	241	2.08	3.54	137^a,b^	127
SEM		18.8	0.29	0.56	11.4	10.1
*p*‐Values						
Thyme		0.564	0.664	0.892	0.559	0.039
Se		0.929	0.521	0.037	0.946	0.534
Thyme × Se		0.751	0.751	0.237	0.045	0.134

^a,b^ Means within a column followed by the same superscript are not significantly different (*p* > 0.05).

SEM: Standard error of mean.

**TABLE 4:** The effects of dietary supplemental thyme and/or selenium on activity of GPx in blood of broiler chickens

Groups		Activity of GPx in blood, μ/L
Thyme		
0		1971
250		2457
Se		
0		1931^a,b^
0.3		2498^a,b^
Thyme	Se	
0	0	1547
250	0	2314
0	0.3	2396
250	0.3	2600
SEM		102
*p*‐Values		
Thyme		0.0714
Se		0.0414
Thyme × Se		0.263

^a,b^ Means within a column followed by the same superscript are not significantly different (*p* > 0.05).

SEM, Standard error of mean.

## 4 DISCUSSION

### 4.1 Production performance

Demir et al. (2005) indicated no differences in BW, FI and FCR of broilers fed diets supplemented with antibiotic growth promoter compared with five herbal feed additives. Attia et al. (2017a, 2017b) concluded that supplementation with TEO (1.0 g/kg) may be effective for growth promotion in broiler chickens reared under hot climate. Najafi and Torki (2010) reported that the broilers fed a thyme‐included diet had significantly better BW and FCR. In contrast to Demir et al. (2005) and Ragaa et al. (2016), reported thyme did not change FCR in the current study. It is found that thyme (Demir & Kilinc, 2008) and thyme oil (Bolukbasi et al., 2006) had no influence on broilers' performance. Similarly, no improvement was detected in poultry performance due to thymol (Lee et al., 2004). However, better WG values in broilers by adding thyme to the drinking water (Hertrampf, 2001) and diet (Hertrampf, 2001; Ragaa et al., 2016) are reported. No significant differences in WG of broilers were observed when a blend of extracts of sage, thyme and rosemary were added to diets (Hernandez et al., 2004). Actually, mechanisms of action of herbal products are not very clearly defined yet. Placha et al. (2014) reported that TEO may improve intestinal barrier integrity in broilers. The crucial role of small intestine villi in nutrient absorption is strongly demonstrated. An improvement in villus height was detected in broilers fed with thyme (1 g/kg) by Ragaa et al. (2016). They also reported that thyme exerts significant positive effects on broiler performance and immunity parameters. A mixture of essential oils of thyme, anise, oregano, carvacrol, yucca extract and cinnamaldehyde could increase villus height and width in broilers infected with *Clostridium perfringens* (Abudabos et al., 2018). Besides, the improvement in broiler BW and WG due to TEO could be attributed to its positive effect on nutrient digestibility, as reported by Langhout (2000). In addition, this positive result could be, at least in part, due to the herbal products' anti‐oxidant and anti‐bacterial effects in the intestine (Nascimento et al., 2000). There are suggestions that herbal additives alter the permeability of the cell membranes and cause destruction of the pathogenic bacteria (Shabaan, 2017). For instance, Tucker (2002) reported that a mixture contains thyme, garlic, anise, cinnamon and rosemary, with no significant effect on the *Lactobacillus* population, strongly inhibited the number of *E. coli*. Diets supplemented with thyme (0.2%) significantly decreased nitrogen excreted in broilers (Shabaan, 2017). This result was attributed to the effect of thyme on microbes which improved FCR and finally increased nitrogen retention. Besides, since the anti‐oxidant activity of thyme was strongly demonstrated (Demir et al., 2005; Gulcin et al., 2004; Hernandez et al., 2004; Lee et al., 2003; Nascimento et al., 2000; Yoon et al., 2007), it could decrease the reactive oxygen species, then decrease protein damage which leads to reduction of total nitrogen in litter (Shabaan, 2017). Definitely, various types of herbal additives (herbal essential oils, herbal extracts, etc.) and supplementation routes (in diet or drinking water) could be, at least in part, probable reasons for different results in different experiments.

On the other hand, it is assumed that the beneficial effects of thyme, oregano, du‐sacch, quiponin and garlic on broiler performance could be, at least in part, related to their stimulatory effects on the pancreas to secrete digestive enzymes or even to their appetite stimulation and anti‐microbial properties due to bio‐active components (Demir et al., 2005; Kamel, 2001). Similarly, thyme, containing carvacrol, may exert stimulatory effects on pancreatic digestive enzymes (Hernandez et al., 2004; Lee et al., 2003), and increase nutrient absorption (e.g., amino acids), which subsequently leads to better carcasses (Shabaan, 2017). Herbs and plant extracts also exert positive stimulation on the immune system and have sedative properties (Demir & Kilinc, 2008). In harmony with Pournazari et al. (2017) and Ragaa et al. (2016), in the current study, the birds received thyme exhibited more WG compared to the control group; this result is attributed to the anti‐oxidants and phenolic components in thyme which may reduce harmful gut microflora and increase amino acid absorption (Demir & Kilinc, 2008; Gulcin et al., 2004; Hernandez et al., 2004; Lee et al., 2003). In addition, since bacteria compete with the host for the uptake of amino acids (Shabaan, 2017), thyme with reduction of harmful bacteria may lead to increased amino acid absorption, which in turn, may lead to better WG.

In line with Edens (1996), Payne and Southern (2005), Peric et al. (2009) and Spears et al. (2003), we did not detect any significant effects of SY on WG in any period of the experiment or in the overall data (*p* > 0.05). In line with our results, Yoon et al. (2007) reported that neither organic nor inorganic Se supplementation had an effect on final BW and average WG of the broiler chickens. Insignificant effect of the source or level of Se on broiler performance is also reported by Payne and Southern (2005). In contrast with our and Yoon et al. (2007) results, Attia et al. (2010) showed that organic Se could improve FCR. In line with our results, Peric et al. (2009) reported a numeric, but not significant, improvement in FCR values for the broilers fed the organic Se compared to Na_2_SeO_3_. The difference in the results may be due to the Se concentration in basal experimental diets and experimental period. Ocak et al. (2008) believe that, generally different effects of feed additives in the different experimental periods may be attributed to diet composition (e.g. protein level), as well as digestive system development and higher feed digestibility.

El‐Slamony et al. (2015) suggested that Se and/or Zn supplementation may improve feed utilization and absorption or feed metabolism. Attia et al. (2010) reported that 0.25 or 0.40 ppm Se significantly decreased FI compared with the control group containing 0.10 ppm Se. In agreement with Yoon et al. (2007), SY supplementation suppressed FI values in growing period and the overall data in the current study (*p* < 0.05). Yoon et al. (2007) showed the bioavailability of organic Se (as SY) was higher than inorganic Se (as Na_2_SeO_3_). El‐Slamony et al. (2015) suggested that Se and/or Zn could result in better feed utilization and FCR, which in turn, may be assumed as results of the reduction in FI in treated groups. Either significant or numeric improvements in production performance parameters (e.g., WG, FCR, …) in the current study and also the results of Choct et al. (2004) and Naylor et al. (2000) showed that there may be an additional requirement (more than NRC, 1994 recommendation) for Se by the faster growing and higher yielding broilers.

### 4.2 Blood variables and blood GPx activity

In the current study, SY significantly decreased uric acid levels. Besides, triglyceride levels significantly decreased in the TEO group compared to the control group at the end of the experimental period. Addition of 0.4 mg/kg Se to broilers diet containing 0.3 mg/kg aflatoxin B1 significantly reduced serum uric acid (Liang et al., 2015). It is shown Se deficiency may result in increased blood uric acid (Sun et al., 2015). There was an interaction (*p* < 0.05) between TEO and SY on blood cholesterol level at the end of the experimental period (d 42); the mean comparison shows that cholesterol concentration in the birds which only received thyme is significantly lower than the control group and two other treatments. No significant difference in cholesterol level was detected between the control group, the birds which only received SY and also the birds which received thyme plus SY (d 42) (*p* > 0.05). Serum labile biochemical parameters may reflect the condition of the organism and the changes happening to it under the influence of internal and external factors. Cholesterol concentration tended to decrease by the addition of thyme, although the changes did not reach statistical significance. Behboudi et al. (2016) reported that broilers' diet supplementation with 0.5 and 1% thyme significantly decreased triglycerides concentration. Reduction in cholesterol and triglycerides of broiler and cholesterol of Leghorn chickens due to using thyme oil and thymol is reported respectively by Case et al. (1995) and Lee et al. (2004). However, in contrast to our results, Bolukbasi et al. (2006) did not report hypocholesterolaemic effects of 100 or 200 mg/kg thyme oil added to broiler diets. Bolukbasi et al. (2006) also reported that dietary thyme oil increased the plasma level of triglycerides, LDL and HDL in broilers. Najafi and Torki (2010) and Toghyani et al. (2010) reported that there is no significant decrease in triglycerides level of broilers treated by thyme. Radwan et al. (2008) reported that the addition of 1% thyme to broiler diet resulted in a marked decrease in plasma total lipids. In line with our results, Abd El‐Hack and Alagawany (2015) mentioned several reports in which TG level decreased due to thyme. Hernandez et al. (2004) stated that thymol, which is found in thyme, could decrease TG levels via increasing lipase and bile production (Hernandez et al., 2004). Besides, thymol may positively affect gut condition, stimulating the secretion of salivary amylase, bile salt and pancreatic enzymes (e.g., trypsin, chymotrypsin and lipase) (Platel & Srinivasan, 2004). Ali et al. (2007) reported that adding thyme to native laying hen diet significantly decreased plasma HDL, total cholesterol, triglycerides and total lipids. Besides, more compatible with our results, Abdulkarimi et al. (2011), Ali et al. (2007) and El‐Ghousein and Al‐Beitawi (2009) reported adding thyme reduced cholesterol and TG levels. It is shown that thyme oil may alter fatty acid compositions of broiler leg and breast (Bolukbasi et al., 2006) and rat brain (Youdim & Deans, 2000). The main components of thyme are phenols, thymol (40%) and carvacrol (15%) (Azaz et al., 2004). Since thyme contains some active components, namely essential oils, tannins, glycosides and saponins, formation of insoluble saponin‐cholesterol complexes in gut may be responsible for depressed cholesterol level due to thyme consumption by birds (European Scientific Cooperative on Phytotherapy, 2003). Garlic inhibits lipogenic enzymes' activity and leads to depressed chicken blood cholesterol (Qureshi et al., 1983). Besides, the reduction of triglycerides and cholesterol noticed due to the thyme could be attributed to the lowering effect of thymol or carvacrol on HMG‐Co A reductase, the rate‐limiting enzyme of cholesterol synthesis (Case et al., 1995; European Scientific Cooperative on Phytotherapy, 2003; Lee et al., 2003). More compatible to our results, Lee et al. (2003) indicated that dietary carvacrol, but not thymol, reduced plasma triglycerides and phospholipids and suggested that carvacrol may have more impact on lipogenesis compared with cholesterol biosynthesis. It seems that further study is needed to clarify the mechanism of hypolipidaemic actions of thyme.

In the present study, SY addition significantly increased blood GPx activity. Selenium concentration in blood and tissue is an important regulator of GPx activity. Se is the principal constituent of GPx enzymes and other reductases; this enzyme protects cells and cell membranes and organelles from oxidation and peroxidation due to free radicals, which are constantly produced in the body (Gajcevic et al., 2009; Rotruck et al., 1973). GPx plays a definite role in the reduction of H_2_O_2_ and organic peroxides to water and corresponding alcohols, which is an important step in preventing the production of reactive oxygen radicals (Kohrl et al., 2000). Se was reported to be required for proper functioning of the GPx enzymes (Rotruck et al., 1973) and also necessary to protect poultry from exudative diathesis and pancreatic fibrosis; then Se was established as a necessary mineral in poultry diet (Payne & Southern, 2005), several researchers stated that supplementation of Se to the Turkey poults or broilers' diet increased plasma Se concentration (Payne & Southern, 2005). Gajcevic et al. (2009) reported an increase in bird blood GPx activity caused by SY (selenomethionine).

Several reasons may influence anti‐oxidative enzyme activity, namely age, tissue type, exposure to stress and the amount of Se in the animal body (Spears et al., 2003); accordingly, it is suggested that the only variable in the experiment, concentration of Se supplemented to birds' diet, could be the reason for higher GPx activity. Besides, a positive correlation between diet Se concentration and GPx activity is reported by Leng et al. (2003).

A couple of researchers reported that Se source or level did not influence plasma GPx activity (Payne & Southern, 2005); however, in line with our result, Yoon et al. (2007) detected higher GPx activity in broilers received SY compared with those that did not receive Se supplementation; in addition, several authors detected a significant increased GPx activity due to supplementation of Se to the birds' diet (Payne & Southern, 2005); the same authors also believe that Se concentration in basal diets could be the reason for these discrepant results. Besides, Spears et al. (2003) reported organic forms of Se (as selenomethionine) may result in higher GPx activity in broilers.

## 5 CONCLUSIONS

It is concluded that dietary supplemental thyme oil (250 mg/kg diet) has a favourable effect on WG values. Se (0.3 mg/kg) decreases blood concentration of uric acid as well as blood GPx activity. An interaction is detected between thyme oil and Se, so that thyme oil may alone (without SY) reduces blood cholesterol concentration.

## CONFLICT OF INTEREST

The authors of this manuscript have no conflict of interest to declare.

## ETHICAL STATEMENT

All experimental protocols adhered to the guidelines, which were approved by, the Animal Ethics Committee of Razi University (Kermanshah, Iran) and were in accordance with the EU standards for the protection of animals and/or feed legislation. The ethical approval number based on the submitted MSc student thesis proposal is: 1394, Anim Sci Dep, 113.

## AUTHOR CONTRIBUTIONS

**Shokufe Noruzi**: Data curation, Formal analysis, Software; **Mehran Torki**: Conceptualization, Investigation, Methodology, Project administration, Resources, Supervision, Validation, Writing review & editing; **Hamed Mohammadi**: Writing review & editing.

### PEER REVIEW

The peer review history for this article is available at https://publons.com/publon/10.1002/vms3.736

## Data Availability

The data that supports the findings of this study are available on the request of readers via the email address provided.
